# Supplementary material for: Global variation in force-of-infection trends for human Taenia solium taeniasis/cysticercosis
Source: eLife. 2022 Aug 19;11:e76988. doi: 10.7554/eLife.76988 (PMC9391040; doi:10.7554/eLife.76988)
Supplement: Supplementary file 1. — †Sensitivity and specificity estimated in a Bayesian framework by Praet et al., 2013; †† sensitivity and specificity based on exposure estimated in a Bayesian framework by Praet et al., 2010; *limited information on the specific protocol used for the copro-antigen assay; * Flórez Sánchez et al., 2013 indicate that this diagnostic is suitable to determine exposure through detection of anti-cysticercus immunoglobulin G (IgG) antibodies and was evaluated in Colombian patients to assess cross-reactions with different infectious agents (Taenia saginata, Hymenolepis nana, Echinococcus sp., Fasciola hepatica, Entamoeba histolytica, Ascaris lumbricoides, Mansonella ozzardi, Treponema pallidum, Cryptococcus neoformans and HIV); ** sensitivity and specificity estimated in a Bayesian framework by Praet et al., 2010; ‡ 95%CIs not provided to inform priors, therefore minimal uncertainty introduced around the sensitivity and specificity estimates to construct priors. Original data for two datasets available (under the Creative Commons Attribution License; CC BY 4.0) from the ‡‡International Livestock Research Institute open-access repository (http://data.ilri.org/portal/dataset/ecozd) referenced in Holt et al., 2016 and ‡‡‡University of Liverpool open-access repository (http://datacat.liverpool.ac.uk/352/) referenced in Fèvre et al., 2017; ⱡ sensitivity and specificity estimated in Fleury et al., 2007. DRC: Democratic Republic of the Congo; LPDR: Lao People’s Democratic Republic; Ag; antigen; Ab: antibody; ELISA: enzyme-linked immunosorbent assay; LLGP-EITB: Lentil lectin-purified glycoprotein enzyme-linked immunoelectrotransfer blot; Ig: immunoglobulin; km: kilometers, PCC: porcine cysticercosis; RCT: randomized controlled trial. [file elife-76988-supp1.docx]

**Supplementary File 1**

| Table S1. Summary of studies included in final analysis and the diagnostic parameters used to set the probabilistic constraints for sensitivity and specificity of each test. | | | | | |  |
| --- | --- | --- | --- | --- | --- | --- |
| **Study author, year and supplementary reference** | **Location, country** | **Diagnostic** | **Sensitivity (%); specificity (%) median (95% confidence intervals given in the literature)** | ***α*, *β* shape parameters to construct each Beta distribution for sensitivity (Se) and specificity (Sp) priors (informed by column 4)** | **Total sample size** | **Study design, sampling strategy and representativeness** |
| Human taeniasis (copro-antigen and antibody) | | | | | | |
| Gomes *et al*. (2002) | Mulungu do Morro, Brazil | Copro-antigen (Ag) enzyme-linked immunosorbent assay (ELISA) (Allan *et al*., 1990)* | 84.5 (61.9 – 98.0)  92.0 (90.0 – 93.8)^†^ | Se: 11.5, 2.11  Sp: 100, 8.7 | 576 | Cross-sectional (CS) community survey. Household (HH)-level **random sampling** from census (6% population); blood sample collected from every consenting HH member (854/900 persons). |
| Mwape *et al*. (2012) | Petauke district, Zambia | Copro-Ag ELISA (Allan *et al*., 1990) | 84.5 (61.9 – 98.0)  92.0 (90.0 – 93.8)^†^ | Se: 11.5, 2.11  Sp: 100, 8.7 | 712 | CS community survey. All villages within 20 km of Kakwiya Rural Health Centre & **all individuals invited to participate**. Kakwiya is a pig keeping community without ongoing sanitation programme(s). |
| Madinga *et al*. (2017) | Kimpese, DRC | Copro-Ag ELISA (Allan *et al*., 1990) | 84.5 (61.9 – 98.0)  92.0 (90.0 – 93.8)^†^ | Se: 11.5, 2.11  Sp: 100, 8.7 | 4599 | CS community survey (baseline data from a community-based intervention study). 24 villages in a 50 km radius around Kimpese city selected based on pre-inclusion criteria. **All eligible** HH & HH members invited to participate. |
| Holt *et al*. (2016) ^‡‡^ | Luang Prabang and Savannakhet Provinces, Lao PDR | rES33-immunoblot (antibody) (Wilkins *et al*., 1999) | 97.6 (94.0 – 99.0)  99.0 (97.2 – 99.0) | Se: 100, 2.46  Sp: 99, 1 | 766 | CS community survey. **Random selection** of 59 villages (split between two provinces), with 15 HHs randomly selected in each village (regardless of pig ownership). One eligible HH member **randomly selected**. Two provinces selected, one upland and one lowland with differing climate, topography, farming systems, ethnicities and socioeconomics (aim to be representative across Lao PDR). |
| **Human cysticercosis (antibody)** | | | | | |  |
| Theis *et al*. (1994) | Bali | Lentil lectin-purified glycoprotein enzyme-linked immunoelectrotransfer blot (LLGP-EITB) (Tsang *et al*., 1989) | 97.0 (95.0 – 1.00)  97.0 (94.0 – 99.0)^††^ | Se: 100, 3.1  Sp: 100, 3.1 | 820 | CS community and clinic-based survey. 746/2410 sera samples collected from **original randomized survey** across 4 subdistricts (intended to be representative of 4 ecological zones in Bali). Additional 74 sera samples collected from epileptic patients at different hospitals/clinics. |
| Gomes *et al*. (2002) | Mulungu do Morro, Brazil | LLGP-EITB (Tsang *et al*., 1989) | 97.0 (95.0 – 1.00)  97.0 (94.0 – 99.0)^††^ | Se: 100, 3.1  Sp: 100, 3.1 | 668 | See previous Gomes *et al*. 2002 |
| Moro *et al*. (2003) | Vichaycocha, Peru | LLGP-EITB (Tsang *et al*., 1989) | 97.0 (95.0 – 1.00)  97.0 (94.0 – 99.0)^††^ | Se: 100, 3.1  Sp: 100, 3.1 | 317 | CS community survey. Single village selected (part of human cystic echinococcosis study) with blood samples taken from consenting villages (317/472). **Limited further info on sampling strategy**. Similar pig: human seroprevalence ratio in this village vs. other endemic Peruvian villages. |
| Lescano *et al*. (2009) | Tumbes, Peru | LLGP-EITB (Tsang *et al*., 1989) | 97.0 (95.0 – 1.00)  97.0 (94.0 – 99.0)^††^ | Se: 100, 3.1  Sp: 100, 3.1 | 738 | CS community survey (baseline data from longitudinal intervention study). **All eligible individuals** selected in 7 (poor, rural) villages these villages identified for longitudinal study due to presence of pig farming/ free-roaming). |
| Jayaraman *et al*. (2011) | Vellore district, India | LLGP-EITB (Tsang *et al*., 1989) | 97.0 (95.0 – 1.00)  97.0 (94.0 – 99.0)^††^ | Se: 100, 3.1  Sp: 100, 3.1 | 1056 | CS community survey. **Representative, random sample** of individuals without history of seizures) across Vellore district. |
| Weka *et al*. (2013) | Jos metropolis, Nigeria | IgG Ab-ELISA (DiagnosticAutomation/ CortezDiagnostic, Inc. (2016)) | 87.8 (79.0 – 93.9)  95.8 (86.0 – 99.0) | Se: 58.4, 8.1  Sp: 26.7, 1.2 | 125 | CS community survey. Pig-keeping HHs in 9 **locations randomly visited** – individuals in each HH responsible for pig husbandry selected. |
| Edia-Asuke *et al*. (2015) | Kaduna metropolis, Nigeria | IgG Ab-ELISA (DiagnosticAutomation/ CortezDiagnostic, Inc. (2016)) | 87.8 (79.0 – 93.9)  95.8 (86.0 – 99.0) | Se: 58.4, 8.1  Sp: 26.7, 1.2 | 296 | CS community survey. **Random selection** of individuals across 4 areas of Kaduna Metropolis. Study area chosen because of pig rearing & pork consumption practices common. |
| Holt *et al*. (2016) ^‡‡^ | Luang Prabang and Savannakhet Provinces, Lao PDR | rT24H-immunoblot (antibody) (Hancock *et al*., 2006) | 96.0 (93.0 – 99.0)  98.0 (96.0 – 1) | Se: 100, 2.5  Sp: 100, 1 | 744 | See previous Holt *et al*. 2016 |
| Flórez Sánchez *et al*. (2013) | 24 departments across Colombia | IgG Ab-ELISA (López *et al*., 1988) | 1 (95% CI not provided)  97.6 (95% CI not provided) | Se: 98,1  Sp: 29.28, 1.08^‡^ | 29360 | CS community survey. **Three-stage clustered random sampling framework**: municipality primary sampling unit & stratified based on socio-economic factors; within each stratum, second sampling unit (SSU) at neighborhood/village level randomly selected; 10 HH within SSU randomly selected and 1 individual from each HH randomly selected. 23/32 departments across Colombia sampled. |
| **Human cysticercosis (antigen)** | | | | | |  |
| Nguekam *et al*. (2003) | Menoua Division, Cameroon | B158/B60 Ag-ELISA; antigen ELISA using monoclonal antibodies vs excretory-secretory glycoproteins of *T. saginata* (Brandt *et al*., 1992; Dorny *et al*., 2000) | 90.0 (80.0 – 99.0)  98.0 (97.0 – 99.0)** | Se: 41.8, 4.6  Sp: 100, 2.04 | 4993 | CS community survey. Three rural communities chosen based on presence of pig farming & free-roaming, and pork consumption**. Sampling not carried out at random**, but on voluntary basis at village level (therefore sample could not be considered representative of general population). |
| Kanobana *et al*. (2011) | Malanga, DRC | B158/B60 Ag-ELISA (Brandt *et al*., 1992; Dorny *et al*., 2000) | 90.0 (80.0 – 99.0)  98.0 (97.0 – 99.0)** | Se: 41.8, 4.6  Sp: 100, 2.04 | 905 | CS community survey. **All eligible villages** invited to participate in study. Target village identified from previous porcine cysticercosis (PCC) survey area (PCC prevalence range from 25 – 40%). |
| Mwape *et al*. (2012) | Petauke district, Zambia | B158/B60 Ag-ELISA (Brandt *et al*., 1992; Dorny *et al*., 2000) | 90.0 (80.0 – 99.0)  98.0 (97.0 – 99.0)** | Se: 41.8, 4.6  Sp: 100, 2.04 | 708 | See previous Mwape *et al*. 2012 |
| Conlan *et al*. (2012) | Four provinces, Lao PDR | B158/B60 Ag-ELISA (Brandt *et al*., 1992; Dorny *et al*., 2000) | 90.0 (80.0 – 99.0)  98.0 (97.0 – 99.0)** | Se: 41.8, 4.6  Sp: 100, 2.04 | 1306 | CS community survey. Four provinces selected representing where all four ethno-linguistic families present (& areas identified for poverty alleviation, rural development and improving pig production). In each province, one district **randomly selected**, with 6 **randomly selected** villages (in dry season). 14 HH were **then randomly selected** & all eligible HH members asked to participate. |
| Sahlu *et al*. (2019) | 60 villages across three provinces in Burkina Faso | B158/B60 Ag-ELISA (Brandt *et al*., 1992; Dorny *et al*., 2000) | 90.0 (80.0 – 99.0)  98.0 (97.0 – 99.0)** | Se: 41.8, 4.6  Sp: 100, 2.04 | 2933 | Prevalence case-control study (for epilepsy & sequalae) using baseline CS component of cluster RCT. One person per concession (several HHs) sampled – up to 80 concessions selected per village. In each concession, one HH **randomly selected**, and 1 HH member **randomly selected** (80 individual per village, serology from first 60). Concession sampling based on the presence of pigs. |
| Wardrop *et al*. (2015) ^‡‡‡^ | Lake Victoria crescent area, Kenya | HP10 Ag-ELISA; antigen ELISA using monoclonal antibodies vs excretory-secretory glycoproteins of *Taenia saginata* (Harrison *et al*., 1989) | 84.8 (74.4 – 95.2)  94 (90.2 – 97.8) ^ⱡ^ | Se : 47.5, 8.5  Sp : 100, 6.4 | 2089 | CS community survey. Area selected because of high risk of zoonotic disease in Lake Victoria crescent. Clustered sampling with 416 HH **randomly selected** (number of HH per sub-location weighted on cattle population). |
| ^†^Sensitivity and specificity estimated in a Bayesian framework by Praet *et al*. (2013); ^††^ sensitivity and specificity based on exposure estimated in a Bayesian framework by Praet *et al*. (2010); *limited information on the specific protocol used for the copro-antigen assay; * *Flórez Sánchez et al. (2013) indicate that this diagnostic is suitable to determine exposure through detection of anti-cysticercus immunoglobulin G (IgG) antibodies and was evaluated in Colombian patients to assess cross-reactions with different infectious agents (Taenia saginata, Hymenolepis nana, Echinococcus sp., Fasciola hepatica, Entamoeba histolytica, Ascaris lumbricoides, Mansonella ozzardi, Treponema pallidum, Cryptococcus neoformans and HIV)*; ** sensitivity and specificity estimated in a Bayesian framework by Praet *et al*. (2010); ^‡^ 95%CIs not provided to inform priors, therefore minimal uncertainty introduced around the sensitivity and specificity estimates to construct priors. Original data for two datasets available (under the [Creative Commons Attribution License; CC BY 4.0](https://creativecommons.org/licenses/by/4.0/)) from the ^‡‡^International Livestock Research Institute open-access repository (<http://data.ilri.org/portal/dataset/ecozd>) referenced in Holt *et al*. (2016) and ^‡‡‡^University of Liverpool open-access repository ([http://datacat.liverpool.ac.uk/352/)](http://datacat.liverpool.ac.uk/352/)%20) referenced in Fèvre *et al*. (2017); ^ⱡ^ sensitivity and specificity estimated in Fleury *et al*. (2007).  DRC: Democratic Republic of the Congo; LPDR: Lao People’s Democratic Republic; Ag; antigen; Ab: antibody; ELISA: enzyme-linked immunosorbent assay; LLGP-EITB: Lentil lectin-purified glycoprotein enzyme-linked immunoelectrotransfer blot; Ig: immunoglobulin; km: kilometers, PCC: porcine cysticercosis; RCT: randomized controlled trial. | | | | | | |
