# Supplementary material for: Global variation in force-of-infection trends for human Taenia solium taeniasis/cysticercosis
Source: eLife. 2022 Aug 19;11:e76988. doi: 10.7554/eLife.76988 (PMC9391040; doi:10.7554/eLife.76988)
Supplement: Supplementary file 2. — * rES38 Ab-immunblot (Wilkins et al., 1999) ** rT24H Ab-ELISA (Hancock et al., 2006) †B158/B60 Ag-ELISA (Brandt et al., 1992; Dorny et al., 2000) †† LLGP-EITB (Tsang et al., 1989) ⱡAb-ELISA confirmed by immunoblot (Sato et al., 2018) ⱡ ⱡCopro-PCR (Sato et al., 2018). DRC: Democratic Republic of the Congo (DRC); Lao PDR: Lao People’s Democratic Republic. [file elife-76988-supp2.docx]

**Supplementary File 2**

| Table S2. Observed prevalence estimates from sub-Saharan Africa, South America, and Asia for studies referring to “hyper-“or “highly-“endemic setting for human taeniasis (HTT) and human cysticercosis (HCC). | | | | | |
| --- | --- | --- | --- | --- | --- |
| **Reference** | **Location** | **Prevalence (%)** | | | |
|  |  | **HTT antibody** | **HTT**  **Copro-Ag (coprology)** | **HCC**  **antibody** | **HCC**  **antigen** |
| **sub-Saharan Africa** | | | | | |
| Mwanjali et al. (2013) | Tanzania | 4.1* | 5.2 (1.1) | 45.3** | 16.7^†^ |
| Madinga et al. (2017) | DRC |  | 23.4 |  |  |
| Mwape et al. (2012) | Zambia |  | 6.3 |  | 5.8^†^ |
| **South America** | | | | | |
| Garcia et al. (2003) | Peru |  | 2.8 | 13.1^††^ |  |
| Moyano et al. (2016) | Peru |  |  | 36.9^††^ |  |
| **Asia** | | | | | |
| Sato et al. (2018) | Lao PDR | 7.2 ^ⱡ^ | 3.1 ^ⱡ ⱡ^ |  |  |
| Okello et al. (2014) | Lao PDR |  | 26.1 | 66.7^††^ |  |
| * rES38 Ab-immunblot (Wilkins et al., 1999); ** rT24H Ab-ELISA (Hancock et al., 2006); ^†^B158/B60 Ag-ELISA (Brandt et al., 1992; Dorny et al., 2000); ^††^ LLGP-EITB (Tsang et al., 1989); ^ⱡ^Ab-ELISA confirmed by immunoblot (Sato et al., 2018); ^ⱡ ⱡ^Copro-PCR (Sato et al., 2018).  DRC: Democratic Republic of the Congo (DRC); Lao PDR: Lao People’s Democratic Republic. | | | | | |
