# Supplementary material for: Global variation in force-of-infection trends for human Taenia solium taeniasis/cysticercosis
Source: eLife. 2022 Aug 19;11:e76988. doi: 10.7554/eLife.76988 (PMC9391040; doi:10.7554/eLife.76988)
Supplement: Supplementary file 3. — For diagnostic methods used see the corresponding study in Supplementary file 1. Seroprevalence results are accompanied by 95% confidence intervals (95% CI) calculated by the Clopper-Pearson exact method. Parameter median posterior estimates are presented with 95% Bayesian credible intervals (95% BCI) and Deviance information criterion (DIC) model fitting scores; * Diagnostic sensitivity and specificity jointly fitted for the Copro-Ag ELISA (Coral-Almeida et al., 2015). † Best-fitting model determined by DIC (jointly-fitted dataset). †† Best-fitting model determined by DIC (individually-fitted dataset). NA = Not applicable; PDR: People’s Democratic Republic. [file elife-76988-supp3.docx]

**Supplementary File 3**

| **Table S3. The deviance information criterion (DIC) and parameter estimates for simple and reversible catalytic models fitted to each observed human taeniasis (antibody and antigen) age-seroprevalence dataset (ordered by decreasing value of all-age seroprevalence).** | | | | | | | | | |
| --- | --- | --- | --- | --- | --- | --- | --- | --- | --- |
| **Dataset;**  **Marker;**  **Country** | **All-age observed sero- prevalence (%)**  **(95% CI)** | **Catalytic model** | **DIC value** | **Diagnostic sensitivity**  **(95% BCI)** | **Diagnostic specificity**  **(95% BCI)** | ***λ* = infection acquisition (*λ_inf_*) or seroconversion (*λ_sero_*) rate,**  **year^-1^**  **(95% BCI)** | **1/*λ* = average time until becoming antibody seropositive (1/ *λ_sero_*) or infected (1/ *λ_inf_*) (years),**  **(95% BCI)** | ***ρ* = infection loss (*ρ_inf_*) or seroreversion (*ρ_sero_*) rate, year^-1^ (95% BCI)** | **1/*ρ* = average time humans remain seropositive (1/ *λ_sero_)* or infected (1/ *λ_inf_*)**  **(years)**  **(95% BCI)** |
| Jointly-fitted datasets – Simple catalytic model* | | | | | | | | | |
| Mwape *et al*. (2012)  Antigen;  Zambia | 6.32  (4.65 – 8.37) | Simple | 52.51 | 0.835  (0.510 –0.976) | 0.951  (0.937 – 0.964) | 0.00085  (0.00009–  0.0023) | 1,170.41  (431.96 – 11,290.49) | NA | NA |
| Gomes *et al*. (2002)  Antigen;  Brazil | 4.51  (2.97 – 6.54) | Simple |  |  |  | 0.00022  (0.00002 – 0.001) | 4,508.79  (975.98 –  53,292.80) | NA | NA |
| Jointly-fitted datasets – Reversible catalytic model* | | | | | | | | | |
| Mwape *et al*. (2012)  Antigen;  Zambia | 6.32  (4.65 – 8.37) | Reversible | 50.42^†^ | 0.824  (0.533 – 0.972) | 0.959  (0.941 – 0.976) | 0.021  (0.0038 – 0.062) | 47.53  (16.22 – 260.75) | 0.768  (0.362 – 0.991) | 1.303  (1.01 – 2.76) |
| Gomes *et al*. (2002)  Antigen;  Brazil | 4.51  (2.97 – 6.54) | Reversible |  |  |  | 0.0096  (0.00072 – 0.032) | 104.31  (31.33 – 1383.94) | 0.731  (0.379 – 0.978) | 1.37  (1.02 – 2.64) |
| Individually-fitted datasets | | | | | | | | | |
| Holt *et al*. (2016)  Antibody;  Lao PDR | 2.49  (1.51 – 3.87) | Simple | 37.01^††^ | 0.982  (0.959 – 0.996) | 0.992  (0.978 – 0.999) | 0.00044  (0.000103 – 0.00082) | 2264.05  (1,215.35 – 9,696.49) | NA | NA |
| Holt *et al*. (2016)  Antibody;  Lao PDR | 2.49  (1.51 – 3.87) | Reversible | 37.87 | 0.978  (0.936 – 0.995) | 0.993  (0.977 – 0.999) | 0.013  (0.0018 – 0.0304) | 74.94  (32.91 – 556.49) | 0.761  (0.376 – 0.987) | 1.31  (1.01 –  2.66) |
| For diagnostic methods used see the corresponding study in Supplementary File 1. Seroprevalence results are accompanied by 95% confidence intervals (95% CI) calculated by the Clopper-Pearson exact method. Parameter median posterior estimates are presented with 95% Bayesian credible intervals (95% BCI) and Deviance information criterion (DIC) model fitting scores;  * Diagnostic sensitivity and specificity jointly fitted for the Copro-Ag ELISA (Allan *et al*. 1990).  ^†^ Best-fitting model determined by DIC (jointly-fitted dataset). ^††^ Best-fitting model determined by DIC (individually-fitted dataset).  NA = Not applicable; PDR: People’s Democratic Republic.. | | | | | | | | | |
