# Supplementary material for: Global variation in force-of-infection trends for human Taenia solium taeniasis/cysticercosis
Source: eLife. 2022 Aug 19;11:e76988. doi: 10.7554/eLife.76988 (PMC9391040; doi:10.7554/eLife.76988)
Supplement: Supplementary file 4. — For diagnostic methods used see the corresponding study in Supplementary file 1. Seroprevalence results are accompanied by 95% confidence intervals (95% CI) calculated by the Clopper-Pearson exact method. Parameter median posterior estimates are presented with 95% Bayesian credible intervals (95% BCI) and Deviance information criterion (DIC) model fitting scores; * Diagnostic sensitivity and specificity for the antibody lentil lectin-purified glycoprotein enzyme-linked immunoelectrotransfer blot (Ab LLGP-EITB) assay (Tsang et al., 1989) were jointly fitted across datasets. ** Diagnostic sensitivity and specificity for the antibody Ab-ELISA (DiagnosticAutomation/CortezDiagnostic (2006)) assay were jointly fitted across datasets. † Best-fitting model determined by DIC (jointly-fitted dataset). †† Best-fitting model determined by DIC (individually-fitted dataset). NA = Not applicable; PDR: People’s Democratic Republic. [file elife-76988-supp4.docx]

**Supplementary File 4**

| **Table S4. The deviance information criterion (DIC) and parameter estimates for simple and reversible catalytic models fitted to each observed human cysticercosis antibody age-seroprevalence dataset (ordered by decreasing value of all-age seroprevalence).** | | | | | | | | | | | |
| --- | --- | --- | --- | --- | --- | --- | --- | --- | --- | --- | --- |
| **Dataset;**  **Country** | **All-age observed sero- prevalence (%)**  **(95% CI)** | **Catalytic model** | **DIC**  **value** | **Diagnostic sensitivity**  **(95% BCI)** | | **Diagnostic specificity**  **(95% BCI)** | | ***λ_sero_* = seroconver-sion rate,**  **year^-1^**  **(95% BCI)** | **1/*λ_sero_* = average time until becoming antibody seropositive (years)**  **(95% BCI)** | ***ρ_sero_* = seroreversion rate, year^-1^ (95% BCI)** | **1/*ρ_sero_* = average time humans remain antibody seropositive (years)**  **(95% BCI)** |
| Jointly fitted datasets – Simple catalytic model* | | | | | | | | | | | |
| Lescano *et al*. (2009);  Peru | 24.66  (21.59 – 27.94) | Simple | 259.32 | 0.969  (0.930 – 0.991) | | 0.929  (0.911 – 0.946) | | 0.00829  (0.0063 – 0.011) | 120.63  (90.91 – 158.73) | NA | NA |
| Moro *et al*. (2003);  Peru | 20.82  (16.48 – 25.71) | Simple |  |  |  |  |  | 0.00311  (0.0015 – 0.0051) | 321.49  (197.84 – 663.30) | NA | NA |
| *J*ayaraman *et al*. (2011);  India | 15.81  (13.66 – 18.16) | Simple |  |  |  |  |  | 0.0032  (0.0022 – 0.0043) | 312.00  (231.28 – 457.19) | NA | NA |
| Theis *et al*. (1994);  Bali | 12.68  (10.48 – 15.16) | Simple |  |  |  |  |  | 0.00348  (0.0018 – 0.0054) | 287.22  (185.16 – 558.35) | NA | NA |
| Gomes *et al*. (2002);  Brazil | 1.64  (0.82 – 2.93) | Simple |  |  |  |  |  | 0.000078  (0.000012 – 0.00035) | 12,892.13  (2,854.80 – 82,689.12) | NA | NA |
| Jointly-fitted datasets – Reversible catalytic model* | | | | | | | | | | | |
| Lescano *et al*. (2009);  Peru | 24.66  (21.59 – 27.94) | Reversible | 156.28^†^ | 0.976  (0.937 – 0.994) | | 0.980  (0.967 – 0.988) | | 0.12  (0.067 – 0.19) | 8.45  (5.20 – 15.03) | 0.38  (0.21 – 0.62) | 2.63  (1.60 – 4.69) |
| Moro *et al*. (2003);  Peru | 20.82  (16.48 – 25.71) | Reversible |  |  |  |  |  | 0.11  (0.0504 – 0.21) | 8.77  (4.77 – 19.82) | 0.501  (0.23 – 0.92) | 2.00  (1.09 – 4.37) |
| Jayaraman *et al*. (2011);  India | 15.81  (13.66 – 18.16) | Reversible |  |  |  |  |  | 0.019  (0.0095 – 0.093) | 52.32  (10.81 – 105.54) | 0.105  (0.042 – 0.56) | 9.51  (1.79 – 23.70) |
| Theis *et al*. (1994);  Bali | 12.68  (10.48 – 15.16) | Reversible |  |  |  |  |  | 0.024  (0.011 – 0.052) | 42.38  (19.47 – 92.51) | 0.16  (0.054 – 0.38) | 6.19  (2.64 – 18.57) |
| Gomes *et al*. (2002);  Brazil | 1.64  (0.82 – 2.93) | Reversible |  |  |  |  |  | 0.000086 (0.000011 – 0.00066) | 11,595.71  (1,519.803 – 91,577.104) | 0.43  (0.098 – 1.49) | 2.32  (0.67 – 10.19) |
| Jointly fitted datasets – Simple catalytic model** | | | | | | | | | | | |
| Edia-Asuke *et al*. (2015);  Nigeria | 14.53  (10.72 – 19.06) | Simple | 38.21^†^ | | 0.872  (0.784 – 0.942) | | 0.974  (0.916 – 0.998) | 0.0044  (0.0018 – 0.0064) | 226.98  (156.27 – 507.04) | NA | NA |
| Weka *et al*. (2013);  Nigeria | 9.60  (5.06 – 16.17) | Simple |  |  |  |  |  | 0.0023  (0.00053 – 0.0046) | 434.95  (217.65 – 1,888.93) | NA | NA |
| Jointly fitted datasets – Reversible catalytic model** | | | | | | | | | | | |
| Edia-Asuke *et al*. (2015);  Nigeria | 14.53  (10.72 – 19.06) | Reversible | 54.62 | | 0.880  (0.791 – 0.943) | | 0.878  (0.846 – 0.919) | 0.0063  (0.00074 – 0.044) | 158.30  (22.54 – 1,356.21) | 5.55  (0.092 – 11.81) | 0.18  (0.08 – 10.88) |
| Weka *et al*. (2013);  Nigeria | 9.60  (5.06 – 16.17) | Reversible |  |  |  |  |  | 0.0027  (0.00033 – 0.019) | 371.93  (53.69 – 3,050.51) | 6.37  (0.86 – 11.27) | 0.16  (0.09 – 1.16) |
| Individually-fitted datasets | | | | | | | | | | | |
| Holt *et al*. (2016);  Lao PDR | 2.96  (1.86 – 4.44) | Simple | 36.62^††^ | 0.964  (0.914 – 0.988) | | 0.986  (0.969 – 0.997) | | 0.00044  (0.000049 – 0.00090) | 2,289.04  (1,111.72 – 20,402.51) | NA | NA |
| Holt *et al*. (2016);  Lao PDR | 2.96  (1.86 – 4.44) | Reversible | 48.34 | 0.963  (0.916 – 0.988) | | 0.973  (0.960 – 0.984) | | 0.00048  (0.000066 – 0.0038) | 2,077.84  (266.53 – 15,092.40) | 1.27  (0.099 – 2.55) | 0.79  (0.39 – 10.02) |
| For diagnostic methods used see the corresponding study in Supplementary File 1. Seroprevalence results are accompanied by 95% confidence intervals (95% CI) calculated by the Clopper-Pearson exact method. Parameter median posterior estimates are presented with 95% Bayesian credible intervals (95% BCI) and Deviance information criterion (DIC) model fitting scores;  * Diagnostic sensitivity and specificity for the antibody lentil lectin-purified glycoprotein enzyme-linked immunoelectrotransfer blot (Ab LLGP-EITB) assay (Tsang *et al*., 1989) were jointly fitted across datasets.  ** Diagnostic sensitivity and specificity for the antibody Ab-ELISA (DiagnosticAutomation/CortezDiagnostic (2006)) assay were jointly fitted across datasets.  ^†^ Best-fitting model determined by DIC (jointly-fitted dataset). ^††^ Best-fitting model determined by DIC (individually-fitted dataset).  NA = Not applicable; PDR: People’s Democratic Republic. | | | | | | | | | | | |
