# Supplementary material for: Global variation in force-of-infection trends for human Taenia solium taeniasis/cysticercosis
Source: eLife. 2022 Aug 19;11:e76988. doi: 10.7554/eLife.76988 (PMC9391040; doi:10.7554/eLife.76988)
Supplement: Supplementary file 5. — For diagnostic methods used see the corresponding study in Supplementary file 1. Seroprevalence results are accompanied by 95% confidence intervals (95% CI) calculated by the Clopper-Pearson exact method. Parameter median posterior estimates are presented with 95% Bayesian credible intervals (95% BCI) and Deviance information criterion (DIC) model fitting scores; * Diagnostic sensitivity and specificity for the B158/B60 Ag-ELISA (Brandt et al., 1992; Dorny et al., 2000). † Best-fitting model determined by DIC (jointly-fitted dataset). †† Best-fitting model determined by DIC (individually-fitted dataset). NA = Not applicable; DRC: Democratic Republic of the Congo; PDR: People’s Democratic Republic. [file elife-76988-supp5.docx]

**Supplementary File 5**

| **Table S5. The deviance information criterion (DIC) and parameter estimates for simple and reversible catalytic models fitted to each observed human cysticercosis antigen age-seroprevalence dataset (ordered by decreasing value of all-age seroprevalence).** | | | | | | | | | |
| --- | --- | --- | --- | --- | --- | --- | --- | --- | --- |
| **Dataset;**  **Country** | **All-age observed sero- prevalence (%)**  **(95% CI)** | **Catalytic model** | **DIC**  **value** | **Diagnostic sensitivity**  **(95% BCI)** | **Diagnostic specificity**  **(95% BCI)** | ***λ_inf_* = infection acquisition rate,**  **year^-1^**  **(95% BCI)** | **1/*λ_inf_* = average time until becoming antigen seropositive (years)**  **(95% BCI)** | ***ρ_inf_* = infection loss rate, year^-1^ (95% BCI)** | **1/*ρ_inf_* = average time humans remain antigen seropositive (years)**  **(95% BCI)** |
| Jointly fitted datasets – Simple catalytic model* | | | | | | | | | |
| Kanobana *et al*. (2011);  DRC | 21.66  (19.01 – 24.49) | Simple | 309.87 | 0.843  (0.768 – 0.916) | 0.996  (0.992 – 0.999) | 0.011  (0.0088 – 0.013) | 92.29  (74.45 – 113.63) | NA | NA |
| Mwape *et al*. (2012);  Zambia | 5.79  (4.19 – 7.77) | Simple |  |  |  | 0.0031  (0.00203 – 0.0045) | 318.56  (222.77 – 463.60) | NA | NA |
| Sahlu *et al*. (2019);  Burkina Faso | 2.45  (1.93 – 3.08) | Simple |  |  |  | 0.00099  (0.00071 – 0.0014) | 1,008.41  (732.52 – 1,409.59) | NA | NA |
| Conlan *et al*. (2012);  Lao PDR | 2.22  (1.49 – 3.17) | Simple |  |  |  | 0.000702  (0.0004 – 0.0011) | 1,424.92  (903.91 – 2,585.53) | NA | NA |
| Nguekam *et al*. (2003);  Cameroon | 0.68  (0.47 – 0.95) | Simple |  |  |  | 0.00017  (0.000078 – 0.00029) | 5,720.002  (3,376.93 – 12,874.48) | NA | NA |
| Jointly fitted datasets – Reversible catalytic model* | | | | | | | | | |
| Kanobana *et al*. (2011);  DRC | 21.66  (19.01 – 24.49) | Reversible | 227.06^†^ | 0.909  (0.810 – 0.967) | 0.999  (0.995 – 0.999) | 0.11  (0.077 – 0.17) | 9.15  (5.91 – 13.03) | 0.330  (0.246 – 0.464) | 3.03  (2.16 – 4.07) |
| Mwape *et al*. (2012);  Zambia | 5.79  (4.19 – 7.77) | Reversible |  |  |  | 0.0044  (0.0027 – 0.0088) | 226.55  (113.85 –375.75) | 0.023  (0.001 – 0.085) | 44.42  (11.72 – 672.09) |
| Sahlu *et al*. (2019);  Burkina Faso | 2.45  (1.93 – 3.08) | Reversible |  |  |  | 0.0016  (0.00091 – 0.0032) | 642.11  (308.19 – 1,106.82) | 0.029  (0.0016 – 0.11) | 34.31  (9.31 – 624.45) |
| Conlan *et al*. (2012);  Lao PDR | 2.22  (1.49 – 3.17) | Reversible |  |  |  | 0.0018  (0.00073 – 0.0034) | 547.64  (298.86 – 1,371.48) | 0.063  (0.0076 – 0.12) | 15.91  (8.62 – 130.78) |
| Nguekam *et al*. (2003);  Cameroon | 0.68  (0.47 – 0.95) | Reversible |  |  |  | 0.00017  (0.000077 – 0.00030) | 4,376.16  (2,617.01 – 7,679.21) | 0.004  (0.00018 – 0.026) | 247.89  (38.62 – 5,600.50) |
| Individually-fitted datasets | | | | | | | | | |
| Wardrop *et al*. (2015);  Kenya | 6.61  (5.57 – 7.76) | Simple | 79.55^††^ | 0.850  (0.735 – 0.927) | 0.944  (0.930 – 0.959) | 0.00054  (0.000053 – 0.0013) | 1,868.83  (803.17 – 18,740.50) | NA | NA |
| Wardrop *et al*. (2015);  Kenya | 6.61  (5.57 – 7.76) | Reversible | 92.25 | 0.854  (0.752 – 0.928) | 0.935  (0.924 – 0.946) | 0.00059  (0.000079 – 0.0045) | 1,692.74  (220.57 – 12,631.65) | 1.68  (0.12 – 4.31) | 0.596  (0.23 – 8.07) |
| For diagnostic methods used see the corresponding study in Supplementary File 1. Seroprevalence results are accompanied by 95% confidence intervals (95% CI) calculated by the Clopper-Pearson exact method. Parameter median posterior estimates are presented with 95% Bayesian credible intervals (95% BCI) and Deviance information criterion (DIC) model fitting scores;  * Diagnostic sensitivity and specificity for the B158/B60 Ag-ELISA (Brandt *et al*., 1992; Dorny *et al*., 2000).  ^†^ Best-fitting model determined by DIC (jointly-fitted dataset). ^††^ Best-fitting model determined by DIC (individually-fitted dataset).  NA = Not applicable; DRC: Democratic Republic of the Congo; PDR: People’s Democratic Republic. | | | | | | | | | |
