# Supplementary material for: Global variation in force-of-infection trends for human Taenia solium taeniasis/cysticercosis
Source: eLife. 2022 Aug 19;11:e76988. doi: 10.7554/eLife.76988 (PMC9391040; doi:10.7554/eLife.76988)
Supplement: Supplementary file 6. — For diagnostic methods used see the corresponding study in Supplementary file 1. DIC score for the reversible model was –401.78. Jointly-fitted diagnostic sensitivity was 0.989 (95%BCI: 0.946–0.999) and specificity was 0.998 (95%BCI: 0.993–0.999). Seroprevalence results are accompanied by 95% confidence intervals (95% CI) calculated by the Clopper-Pearson exact method. Parameter median posterior estimates are presented with 95% Bayesian credible intervals (95% BCI) and Deviance information criterion (DIC) model fitting scores. [file elife-76988-supp6.docx]

**Supplementary File 6**

| **Table S6. The deviance information criterion (DIC) and parameter estimates for the reversible catalytic model jointly fitted (for diagnostic sensitivity and specificity) to the observed human cysticercosis antibody age-seroprevalence for each available department in Colombia (n=23, ordered by decreasing value of all-age seroprevalence).** | | | | | |
| --- | --- | --- | --- | --- | --- |
| **Department**  **(sample size, n)** | **All-age observed seroprevalence (%) (95% CI)** | ***λ_sero_* = seroconversion rate,**  **year^-1^**  **(95% BCI)** | **1/*λ_sero_* = average time until becoming antibody seropositive (years)**  **(95% BCI)** | ***ρ_sero_* = seroreversion rate, year^-1^**  **(95% BCI)** | **1/*ρ_sero_* = average time humans remain antibody seropositive (years)**  **(95% BCI)** |
| Vaupés (1,140) | 38.68 (35.85 – 41.58) | 0.065 (0.036 – 0.16) | 15.38 (6.24 – 27.53) | 0.095 (0.046 – 0.25) | 10.47 (3.99 – 21.77) |
| Amazonas (1,210) | 21.74 (19.44 – 24.17) | 0.063 (0.013 – 0.19) | 15.60 (5.24 – 74.20) | 0.22 (0.032 – 0.69) | 4.53 (1.44 – 30.78) |
| Cundinamarca (891) | 14.37 (12.13 – 16.84) | 0.049 (0.018 – 0.16) | 20.45 (6.06 – 55.00) | 0.29 (0.11 – 0.93) | 3.44 (1.08 – 9.34) |
| La Guajira (1,270) | 13.62 (11.78 – 15.63) | 0.059 (0.0097 – 0.13) | 17.09 (7.43 – 102.33) | 0.38 (0.054 – 0.89) | 2.64 (1.12 – 18.60) |
| San Andrés (1,230) | 12.36 (10.57 – 14.33) | 0.026 (0.012 – 0.047) | 38.56 (21.32 – 83.26) | 0.19 (0.084 – 0.32) | 5.40 (3.08 – 11.87) |
| Antioquia (1,291) | 12.01 (10.28 – 13.90) | 0.093 (0.0082 – 0.13) | 10.70 (7.80 – 121.99) | 0.71 (0.050 – 0.85) | 1.41 (1.18 – 19.91) |
| Cesar (1,270) | 11.89 (10.16 – 13.80) | 0.023 (0.010 – 0.087) | 43.77 (11.54 – 96.23) | 0.17 (0.068 – 0.63) | 5.99 (1.58 – 14.60) |
| Cauca (1,270) | 11.18 (9.50 – 13.04) | 0.032 (0.010 – 0.071) | 31.23 (14.13 – 97.66) | 0.26 (0.074 – 0.54) | 3.90 (1.86 – 13.55) |
| Magdalena (1,260) | 9.84 (8.25 – 11.62) | 0.033 (0.013 – 0.054) | 30.56 (18.48 – 74.19) | 0.30 (0.12 – 0.47) | 3.37 (2.12 – 8.31) |
| Atlántico (1,280) | 9.06 (7.55 – 10.77) | 0.025 (0.013 – 0.57) | 40.54 (17.47 – 77.42) | 0.23 (0.13 – 0.57) | 4.30 (1.75 – 7.54) |
| Nariño (1,264) | 6.33 (5.05 – 7.82) | 0.021 (0.0088 – 0.048) | 48.14 (20.87 – 113.25) | 0.32 (0.13 – 0.72) | 3.11 (1.39 – 7.42) |
| Valle Del Cauca (1,260) | 4.92 (3.79 – 6.26) | 0.021 (0.0024 – 0.072) | 47.67 (13.92 – 408.47) | 0.41 (0.036 – 1.39) | 2.44 (0.72 – 27.91) |
| Tolima (1,270) | 4.65 (3.56 – 5.95) | 0.016 (0.0074 – 0.031) | 62.27 (32.59 – 134.42) | 0.34 (0.17 – 0.59) | 2.91 (1.70 – 5.95) |
| Meta (1,262) | 4.36 (3.30 – 5.64) | 0.015 (0.0047 – 0.041) | 66.32 (24.27 – 212.47) | 0.34 (0.11 – 0.82) | 2.91 (1.22 – 9.51) |
| Boyacá (1,270) | 4.02 (3.00 – 5.25) | 0.0039 (0.0011 – 0.028) | 256.18 (35.80 – 872.09) | 0.091 (0.0068 – 0.70) | 10.95 (1.43 – 146.97) |
| Bogotá D.C (850) | 3.53 (2.39 – 5.00) | 0.011 (0.0015 – 0.027) | 88.55 (36.77 – 665.86) | 0.34 (0.030 – 0.66) | 2.91 (1.52 – 32.81) |
| Huila (1,280) | 3.43 (2.51 – 4.59) | 0.0047 (0.0010 – 0.032) | 212.91 (31.19 – 977.68) | 0.14 (0.0054 – 0.87) | 7.36 (1.15 – 183.64) |
| Casanare (1,252) | 2.80 (1.95 – 3.87) | 0.0049 (0.0010 – 0.015) | 204.76 (65.61 – 966.12) | 0.19 (0.023 – 0.51) | 5.34 (1.97 – 43.56) |
| Guaviare (1,220) | 2.70 (1.87 – 3.78) | 0.0072 (0.0019 – 0.026) | 139.16 (38.01 – 539.23) | 0.27 (0.068 – 1.01) | 3.49 (0.99 – 14.73) |
| Santander (1,270) | 2.52 (1.73 – 3.54) | 0.0098 (0.0010 – 0.029) | 102.34 (34.60 – 996.14) | 0.46 (0.018 – 1.070) | 2.17 (0.94 – 55.69) |
| Quindio (1,260) | 2.22 (1.48 – 3.20) | 0.0051 (0.0019 – 0.014) | 197.21 (72.80 – 537.87) | 0.25 (0.098 – 0.58) | 4.06 (1.74 – 10.20) |
| Risaralda (1,270) | 1.39 (0.78 – 2.13) | 0.0016 (0.00029 – 0.0081) | 609.19 (123.15 – 3,425.94) | 0.14 (0.0067 – 0.58) | 6.95 (1.71 – 148.42) |
| Caldas (1,260) | 0.48 (0.17-1.03) | 0.00079 (0.000045 – 0.0045) | 1,263.06 (224.61 – 22,321.43) | 0.30 (0.054 – 0.85) | 3.39 (1.18 – 18.47) |
| For diagnostic methods used see the corresponding study in Supplementary File 1. DIC score for the reversible model was -401.78. Jointly-fitted diagnostic sensitivity was 0.989 (95%BCI: 0.946 – 0.999) and specificity was 0.998 (95%BCI: 0.993 – 0.999). Seroprevalence results are accompanied by 95% confidence intervals (95% CI) calculated by the Clopper-Pearson exact method. Parameter median posterior estimates are presented with 95% Bayesian credible intervals (95% BCI) and Deviance information criterion (DIC) model fitting scores. | | | | | |
