# Supplementary material for: Global variation in force-of-infection trends for human Taenia solium taeniasis/cysticercosis
Source: eLife. 2022 Aug 19;11:e76988. doi: 10.7554/eLife.76988 (PMC9391040; doi:10.7554/eLife.76988)
Supplement: Supplementary file 7. — For diagnostic methods used see the corresponding study in Supplementary file 1. DIC score for the simple model was –442.7. Jointly-fitted diagnostic sensitivity was 0.987 (95%BCI: 0.966–0.999) and specificity was 0.980 (95%BCI: 0.975–0.984). Seroprevalence results are accompanied by 95% confidence intervals (95% CI) calculated by the Clopper-Pearson exact method. Parameter median posterior estimates are presented with 95% Bayesian credible intervals (95% BCI) and Deviance information criterion (DIC) model fitting scores. [file elife-76988-supp7.docx]

**Supplementary File 7.**

| **Table S7. The deviance information criterion (DIC) and parameter estimates for the simple catalytic model jointly fitted (for diagnostic sensitivity and specificity) to the observed human cysticercosis antibody age-seroprevalence for each available department in Colombia (n = 23, ordered by decreasing value of all-age seroprevalence**). | | | |
| --- | --- | --- | --- |
| **Department**  **(sample size, n)** | **All-age observed seroprevalence (%)**  **(95% CI)** | ***λ_sero_* = seroconversion rate, year^-1^**  **(95% BCI)** | **1/*λ_sero_* = average time until becoming antibody seropositive (years)**  **(95% BCI)** |
| Vaupés (1,140) | 38.68 (35.85 – 41.58) | 0.014 (0.013 – 0.016) | 69.49 (62.75 – 77.02) |
| Amazonas (1210) | 21.74 (19.44 –24.17) | 0.0066 (0.0057 – 0.0075) | 151.98 (133.29 – 174.86) |
| Cundinamarca (891) | 14.37 (12.13 – 16.84) | 0.0034 (0.0027 – 0.0042) | 292.03 (246.92 – 366.08) |
| La Guajira (1270) | 13.62 (11.78 – 15.63) | 0.0036 (0.00029 – 0.0042) | 281.41 (236.63 – 340.74) |
| San Andrés (1230) | 12.36 (10.57 – 14.33) | 0.0029 (0.0024 – 0.0035) | 343.07 (283.07 – 419.36) |
| Antioquia (1291) | 12.01 (10.28 –13.90) | 0.0027 (0.0022 – 0.0033) | 366.35 (306.56 – 453.58) |
| Cesar (1270) | 11.89 (10.16 – 13.80) | 0.0029 (0.0026 – 0.0035) | 342.57 (282.18 – 424.10) |
| Cauca (1270) | 11.18 (9.50 – 13.04) | 0.0025 (0.0020 – 0.0031) | 399.19 (319.87 – 494.95) |
| Magdalena (1260) | 9.84 (8.25 – 11.62) | 0.0023 (0.0018 – 0.0028) | 439.17 (353.09 – 565.11) |
| Atlántico (1280) | 9.06 (7.55 – 10.77) | 0.0021 (0.0015 – 0.0026) | 485.98 (379.09 – 651.88) |
| Nariño (1264) | 6.33 (5.05 – 7.82) | 0.0012 (0.00080 – 0.0016) | 834.70 (1242.79 – 613.61) |
| Valle Del Cauca (1260) | 4.92 (3.79 – 6.26) | 0.00075 (0.00043 – 0.0011) | 1,327.11 (884.23 – 2,326.84) |
| Tolima (1270) | 4.65 (3.56 – 5.95) | 0.00070 (0.00037 – 0.0011) | 1,437.40 (918.07 – 2,736.72) |
| Meta (1262) | 4.36 (3.30 – 5.64) | 0.00067 (0.00036 – 0.0011) | 1,485.96 (919.29 – 2,825.13) |
| Boyacá (1270) | 4.02 (3.00 – 5.25) | 0.00060 (0.00029 – 0.00095) | 1,670.93 (1,052.29 – 3,441.97) |
| Bogotá D.C (850) | 3.53 (2.39 – 5.00) | 0.00041 (0.00013 – 0.00080) | 2,461.14 (1,250.71 – 7,784.22) |
| Huila (1280) | 3.43 (2.51 – 4.59) | 0.00046 (0.00018 – 0.00079) | 2,174.64 (1,261.06 – 5,466.00) |
| Casanare (1252) | 2.80 (1.95 – 3.87) | 0.00026 (0.000054 – 0.00057) | 3,786.45 (1,766.88 – 91,388.13) |
| Guaviare (1220) | 2.70 (1.87 – 3.78) | 0.00025 (0.000043 – 0.00055) | 3,994.80 (1,807.03 – 23,180.19) |
| Santander (1270) | 2.52 (1.73 – 3.54) | 0.00018 (0.000033 – 0.00046) | 5,065.60 (2,196.77 – 29,890.66) |
| Quindio (1260) | 2.22 (1.48 – 3.20) | 0.00011 (0.000015 – 0.00033) | 9,210.03 (3,062.74 – 66,454.01) |
| Risaralda (1270) | 1.39 (0.78 – 2.13) | 0.000055 (0.000012 – 0.00019) | 1,8304.61 (5,200.37 – 85,265.30) |
| Caldas (1260) | 0.48 (0.17 – 1.03) | 0.000031 (0.000011 – 0.00011) | 32,772.10 (8,874.67 – 91,388.13) |
| For diagnostic methods used see the corresponding study in Supplementary File 1. DIC score for the simple model was –442.7. Jointly-fitted diagnostic sensitivity was 0.987 (95%BCI: 0.966 – 0.999) and specificity was 0.980 (95%BCI: 0.975 – 0.984).  Seroprevalence results are accompanied by 95% confidence intervals (95% CI) calculated by the Clopper-Pearson exact method. Parameter median posterior estimates are presented with 95% Bayesian credible intervals (95% BCI) and Deviance information criterion (DIC) model fitting scores. | | | |
