# Supplementary material for: Global variation in force-of-infection trends for human Taenia solium taeniasis/cysticercosis
Source: eLife. 2022 Aug 19;11:e76988. doi: 10.7554/eLife.76988 (PMC9391040; doi:10.7554/eLife.76988)
Supplement: Supplementary file 8. — Adapted from Behrend et al., 2020. Full formulation of the principles: Don't do it alone. Engage stakeholders throughout, from the formulation of questions to the discussions on the implications of the findings. Reproducibility is key! Prepare and make available (preferably open-source) a complete technical documentation of all model code, mathematical formulas, assumptions and their justification, allowing others to reproduce the model. Model calibration, goodness-of-fit and validation are fundamental processes of scientific modelling. All data used should be described in sufficient detail to allow the reader to assess the type and quality of these analyses. When using data by reference, use Principle 2. Communicating uncertainty is a hallmark of good modelling practice. Perform a sensitivity analysis of all key parameters, and for each paper reporting model predictions include an uncertainty assessment of those model outputs within the paper. Model outcomes should be articulated in the form of testable hypotheses. This allows comparison with other models and future events as part of the ongoing cycle of model improvement. Incorporating prior work by reference in the paper is sufficient. Write what relevant information is contained in the prior work; then note its reference number in the summary table. Please verify that the whole chain of steps in referenced work is actually complete and up-to-date. [file elife-76988-supp8.docx]

**Supplementary File 8**

**Table S8. PRIME-NTD (Policy-Relevant Items for Reporting Models in Epidemiology of Neglected Tropical Diseases) Summary. Adapted from Behrend *et al*. 2020.**

| Principle | What has been done to  satisfy the principle? | Where in the manuscript is this described? |
| --- | --- | --- |
| 1. **Stakeholder engagement** | Engagement with the *Instituto Nacional de Salud* in Colombia for access to a large dataset, and continued engagement with the stakeholders throughout the analysis & manuscript development. | Intro (pg.5); Discussion (pg.24-25). |
| 1. **Complete model documentation** | Full model description in the manuscript and all model/ fitting code available on the Github link: [*https://github.com/mrc-ide/human_tsol_FoI_modelling*](https://github.com/mrc-ide/human_tsol_FoI_modelling) | Methods (pg. 27-29) and “code availability” statement. |
| 1. **Complete description of data used** | In-depth description of datasets and age-(sero)prevalence aggregated data available. | Methods (pg. 27), Supplementary material tables (pg. 13-15), and “data availability statement”. |
| 1. **Communicating uncertainty** | Methods for generating uncertainty associated with fitted model parameters are described, and Bayesian credible intervals presented in the results. Uncertainty is also interpreted in the Discussion. | Methods (pg. 28-29; including specifying prior distributions to sample uncertainty in Table 5.), throughout Results section, Discussion (pg. 23-24) |
| 1. **Testable model outcomes** | No formal model validation exercises performed; however fitted parameter estimates discussed in terms of literature estimates. | Discussion (pg. 23) |

Full formulation of the principles:

1. Don't do it alone. Engage stakeholders throughout, from the formulation of questions to the discussions on the implications of the findings.
2. Reproducibility is key! Prepare and make available (preferably open-source) a complete technical documentation of all model code, mathematical formulas, assumptions and their justification, allowing others to reproduce the model.
3. Model calibration, goodness-of-fit and validation are fundamental processes of scientific modelling. All data used should be described in sufficient detail to allow the reader to assess the type and quality of these analyses. When using data by reference, use Principle 2.
4. Communicating uncertainty is a hallmark of good modelling practice. Perform a sensitivity analysis of all key parameters, and for each paper reporting model predictions include an uncertainty assessment of those model outputs within the paper.
5. Model outcomes should be articulated in the form of testable hypotheses. This allows comparison with other models and future events as part of the ongoing cycle of model improvement.

Incorporating prior work by reference in the paper is sufficient. Write what relevant information is contained in the prior work; then note its reference number in the summary table. Please verify that the whole chain of steps in referenced work is actually complete and up-to-date.
